# 

**Published:** 1851-07

**Authors:** 


### CONTENTS.

### JOURNAL.

| | Page. |
|---|---|
| Article I.—Treatment of Dental Caries, complicated with Disorders of the Pulp and Peridental Membrane—No. II. By Robert Arthur, D. D. S | 405 |
| Article II.—On the Inexpediency and Impracticability of Teaching Dental Surgery in Medical Colleges. By Elisha Townsend, D. D. S. | 420 |
| Article III.—Iodine in the Treatment of Dental Periostitis and Diseases of the Gums. By James North, M. D., D. D. S. | 437 |
| Article IV.—Cheap Artificial Teeth, and Cheap Dental Operations in England, | 441 |
| Article V.—Filling Teeth over Exposed Nerves. By W. H. Elliot. D. D. S | 445 |
| Article VI.—Morbid Sympathies of the Teeth. By John A. Johns, M. D., D. D. S | 447 |
| Article VII.—Medical Ethics, | 453 |
| Article VIII.—On the Comparative Advantages of Wide and Narrow Plates for Artificial Teeth. By J. W. Crane, M. D. | 458 |
| Article IX.—Valedictory Address delivered to the Graduates of the Baltimore College of Dental Surgery, at the Commencement, held March 28th, 1851. By Eleazar Parmly, M. D. | 460 |

### SELECTED ARTICLES.

| | |
|---|---|
| Article X.—On Filling Teeth. By James Taylor, M. D. | 478 |
| Article XI.—On Ozena. By M. Max-Simon | 487 |

*Contents.*

PAGE

ARTICLE XII.—An Essay upon Clasps for Retaining Partial Sets of Artificial Teeth in the Mouth. By C. A. Dubouchet, M. D. 488
ARTICLE XIII.—Extensive Injury to the Jaw, following the Removal of an Incisor Tooth by the Key Instrument—Remarks on the Extraction of Deciduous Teeth. By Thomas Underwood, Esq........................................ 492
ARTICLE XIV.—Necrosis of the Inferior Maxillary,............... 494
ARTICLE XV.—Disease of the Antrum of Highmore. By R. D. Couch, Esq., M. R. C. S. L................ ............ 497
ARTICLE XVI.—Diseased Temporal Bone,...................... 499
ARTICLE XVII.—Hemorrhage from the Tongue, successfully treated with Tincture of Matico,.............................. 501
ARTICLE XVIII.—Remarks on Mr. Stevenson's comment on Dr. Marshall Hall's view of the Vis Nervosa, and on a case of great loss of nervous power resulting from a carious tooth. By J. L. Levison, Esq.......................... .... 503
ARTICLE XIX.—Head-ache; Suppuration about the Left Ear; Disease of the Temporal Bone; Abscess in the Brain; and remarks. By E. L. Ormerod, M. B...................... 505
ARTICLE XX.—On the Teeth as an Indication of the Progressive Improvement of the Human Race,........................ 508
ARTICLE XXI.—Results of the Use of Chloroform in Nine Thousand Cases at St. Bartholomew's Hospital. By Mr. Skey.... 529
ARTICLE XXII.—A Report of Two Cases of Sloughing Phagedena. By Joseph Drew, M. B............................... 532
ARTICLE XXIII.—New Method of Remedying the Accidents Caused by Chloroform. By M. Ricord...................... 539
ARTICLE XXIV.—Rhinoplastic Operation...................... 541
ARTICLE XXV.—Cancer of the Tongue—Division of the Gustatory Nerve.............................................. 545
ARTICLE XXVI.—Spontaneous Collapse of the Walls of the Antrum..... ........................................ 547
ARTICLE XXVII.—Caries of the Petrous Portion of the Temporal Bone................................................ 548
ARTICLE XXVIII.—The Use and Abuse of Arsenious Acids as an Agent for Destroying the Dental Pulp and Nerve, Curing the Tooth-ache. By A. C. Castle, M. D................. 549
ARTICLE XXIX.—On the Administration of Chloroform......... 551
ARTICLE XXX.—Dislocation of the Lower Jaw................ 554

### EDITORIAL DEPARTMENT.

Dr. Gardette and his Proposition, once more,.................... 557
Neuralgia................................ ...................... 561
Epulis,........................................................ 561
Complimentary Letters,......................................... 562
Compound Lever Forceps,....................................... 562
Antidote to Arsenic,........................................... 562
Dr. J. V. C. Smith,............................................ 563
Phrenology,.................................................... 563
Twelfth Annual Meeting of the American Society of Dental Surgeons,........................................................ 564
Completion of the First Volume of the New Series,.............. 564
Chevalier's Drill Stock,....................................... 564

